# MicroRNAs targeting VEGF are related to vascular dysfunction in preeclampsia

**DOI:** 10.1042/BSR20210874

**Published:** 2021-08-12

**Authors:** Isabel Witvrouwen, Dominique Mannaerts, Jessica Ratajczak, Evi Boeren, Ellen Faes, Amaryllis H. Van Craenenbroeck, Yves Jacquemyn, Emeline M. Van Craenenbroeck

**Affiliations:** 1 Research Group Cardiovascular Diseases, GENCOR, University of Antwerp, Antwerp, Belgium; 2 Department of Cardiology, Antwerp University Hospital, Edegem, Belgium; 3 Research Group ASTARC, Antwerp Surgical Training, Anatomy and Research Centre, University of Antwerp, Antwerp, Belgium; 4 Department of Obstetrics and Gynaecology, Antwerp University Hospital, Edegem, Belgium; 5 Laboratory of Experimental Medicine and Paediatrics, University of Antwerp, Antwerp, Belgium; 6 Department of Nephrology, University Hospitals Leuven, Leuven, Belgium

**Keywords:** angiogenesis, arterial stiffness, endothelial functionlial, microRNA, oxidative stress, preeclampsia

## Abstract

In preeclampsia (PE), pre-existent maternal endothelial dysfunction leads to impaired placentation and vascular maladaptation. The vascular endothelial growth factor (VEGF) pathway is essential in the placentation process and VEGF expression is regulated through post-transcriptional modification by microRNAs (miRNAs). We investigated the expression of VEGF-related circulating miR-16, miR-29b, miR-126, miR-155 and miR-200c in PE vs healthy pregnancies (HPs), and their relation with vascular function, oxidative stress (OS) and systemic inflammation. In this case–control study, 24 women with early PE (<34 weeks) were compared with 30 women with HP. Circulating microRNA levels (RT-qPCR), OS and systemic inflammation were assessed in plasma samples (PE 29.5 vs HP 25.8 weeks) and related to extensive *in vivo* vascular function (flow-mediated dilatation (FMD), modified FMD (mFMD), carotid-femoral pulse wave velocity (CF-PWV), heart rate corrected augmentation index (AIx75) and reactive hyperemia index (RHI)). FMD, CF-PWV, AIx75 and RHI were all significantly impaired in PE (*P*<0.05). PE patients had reduced levels of miR-16 (5.53 ± 0.36 vs 5.84 ± 0.61) and increased levels of miR-200c (1.34 ± 0.57 vs 0.97 ± 0.68) (*P*<0.05). Independent of age and parity, miR-16 was related to impaired FMD (β 2.771, 95% C.I.: 0.023–5.519, *P*=0.048) and mFMD (β 3.401, 95% C.I.: 0.201–6.602, *P*=0.038). Likewise, miR-200c was independently associated with CF-PWV (β 0.513, 95% C.I.: 0.034–0.992, *P*=0.036). In conclusion, circulating levels of miR-16 were lower in PE, which correlated with impaired endothelial function. Circulating miR-200c was increased in PE and correlated with higher arterial stiffness. These findings suggest a post-transcriptional dysregulation of the VEGF pathway in PE and identify miR-16 and miR-200c as possible diagnostic biomarkers for PE.

## Introduction

MicroRNAs (miRNAs) are small non-coding RNA molecules that regulate gene expression at post-transcriptional level [1]. Since they are involved in virtually every biological process, aberrant miRNA expression contributes to disease processes through dysregulation of essential signaling pathways [2]. During pregnancy, the placenta expresses many essential miRNAs, which can be secreted into the maternal circulation packed in exosomes, microvesicles or bound to stabilizing proteins. These miRNAs play important roles in placental development, immunomodulation and uteroplacental and maternal vascular adaptation to pregnancy [3,4].

Preeclampsia (PE) is a pregnancy-specific complication characterized by arterial hypertension in combination with organ involvement such as proteinuria, thrombocytopenia, kidney and liver failure and in severe cases cerebral involvement resulting in eclampsia [5]. The exact pathophysiology of PE remains elusive, but extensive research has proven an indispensable role of pre-existent maternal endothelial dysfunction resulting in disturbed uteroplacental and peripheral vascular adaptation to pregnancy [6]. Optimal vascular adaption to pregnancy requires a complex interaction between different players, with a crucial role of the vascular endothelial growth factor (VEGF) pathway [7–9]. Next, placental growth factor (PlGF), produced by the placenta, is an increasingly important molecule in the prediction, diagnosis and treatment of PE. Low circulating PlGF in combination with high levels of soluble fms-like tyrosine kinase-1 (sFlt-1, the soluble form of the VEGF-1 receptor) is known to precede the manifestation of clinical disease in PE [10,11]. Although underlying vascular disease is present during PE and patients who have had PE are known to have an increased risk of cardiovascular disease later in life, the culprit in the active disease state is the dysfunctional placenta. An abnormal placentation process in the beginning of pregnancy results in an ischemic placenta that releases anti-angiogenic factors (sFlt-1), reactive oxygen species and deregulated concentrations of miRNAs into the maternal circulation. These factors affect the maternal endothelium, resulting in even more endothelial dysfunction and arterial stiffness [12].

VEGF is an important regulator of the placentation process since it regulates angiogenesis and plays a crucial role in the growth of vascular endothelial cells (ECs), the production of placental blood vessels and the promotion of vessel permeability [8,9]. Controversy exists on circulating VEGF levels during healthy pregnancy (HP) vs PE [13–16]. VEGF cannot be considered a reliable predictive marker for PE, hence our approach to study the level of circulating miRNAs that are known to be established regulators of VEGF expression.

*Placental* studies show dysregulated miRNA expression in PE, which renders them potential new therapeutic targets [17]. However, with regard to prediction, diagnosis and monitoring of PE, *circulating* miRNA levels are of greater interest. Therefore, in the present study, we chose to investigate the expression of five (miR-16, miR-29b, miR-126, miR-155, miR-200c) circulating miRNAs involved in the VEGF signaling pathway and their relation to vascular function and oxidative stress (OS) in HP vs PE.

## Materials and methods

### Study population and design

Thirty women with HP and 24 women with early PE (gestational age < 34 weeks) admitted to the maternal intensive care unit were included between November 2015 and June 2017. PE was defined according to the revised ISSHP definition [5].

Exclusion criteria were (gestational) diabetes, multiple pregnancies, fetal malformations, hypercholesterolemia, chronic kidney disease, autoimmune disorders, connective tissue diseases or use of acetylsalicylic acid. Since the Antwerp University Hospital serves as a tertiary referral centre, most women were already initiated on antihypertensive medication, low molecular weight heparin and MgSO_4_ at the time of referral and inclusion.

HP women were free from medication and did not have a history of PE, (pregnancy-induced) hypertension, cardiovascular disease or other chronic conditions.

**Figure 1:**
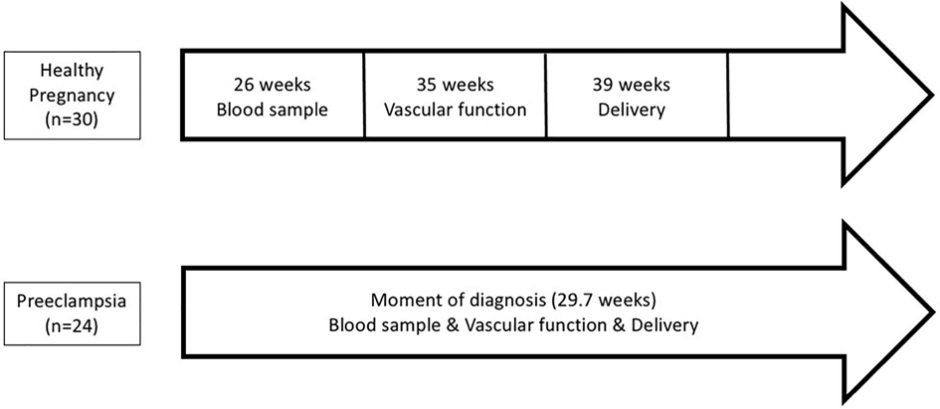
Timeline representing timing of measurements in HP vs PE

Blood samples (22–36 weeks (average: 25.8 weeks)) and vascular function measurements (33–36 weeks (average: 34.7 weeks)) were performed between 22 and 36 weeks in women with HP. Women with PE were included at the time of diagnosis (25–33 weeks; average: 29.7 weeks) (Figure 1). In PE, proteinuria was detected and quantified on a 24-h urine collection.

The present study (ENDOPREG study, Belgian number: B300201524783) complied with the Declaration of Helsinki and was approved by The Research and Ethics committee of the Antwerp University Hospital. Written informed consent was obtained from each participant.

### OS and systemic inflammation measurements

During HP, blood samples for superoxide (O_2_^•−^), neutrophil to lymphocyte ratio (NLR) and mean platelet volume (MPV) were taken at approx. 22–36 weeks (average: 25.8 weeks). In PE, all measurements were performed at diagnosis. Peripheral blood was collected by venepuncture using ethylenediaminetetraacetic acid (EDTA) tubes for the quantification of MPV and NLR. The EDTA tubes were analyzed using an ADVIA 120 Hematology System (Siemens Healthcare®, Germany). Blood concentration of superoxide was determined using electron paramagnetic resonance as previously described [18].

### Vascular function measurements

A comprehensive, non-invasive assessment of endothelial-dependent vasodilation (flow-mediated dilatation (FMD), modified FMD (mFMD) and low-flow mediated constriction (L-FMC) of the brachial artery; Aloka ProSound Alpha, Hitachi), microvascular endothelial function (reactive hyperemia index (RHI), EndoPAT 2000 system, Itamar-Medical, Israel), and large artery stiffness pulse wave velocity and pulse wave analysis (respectively carotid-femoral pulse wave velocity (CF-PWV) and heart rate corrected augmentation index (AIx75), Sphygmocor, AtCor Medical) was performed. All measurements were done in a standardized manner by experienced investigators (IG, TS, DM and EF) as previously published [6,19]. Measurements in HP and PE groups were performed at the same arm and at approximately the same time of day.

### miRNA analysis

A miRNA panel consisting of five miRNAs (miR-16, miR-29b, miR-126, miR-155, miR-200c) involved in angiogenesis and PE pathogenesis was quantified in plasma samples using RT-qPCR. Briefly, whole blood was collected in EDTA tubes. Samples were centrifuged within 30 min after collection (1500×***g***, 15 min) at room temperature and stored at −80°C. Plasma samples were thawed on ice and centrifuged for 10 min (4°C, 16000×***g***). RNA enriched for small RNAs (including miRNAs) was isolated using the mirVana Paris Kit (Thermo Fisher). Reverse transcription and pre-amplification were performed using TaqMan miRNA primers (Thermo Fisher) and multiplex qPCR was done in a CFX96 thermal cycler (Bio-Rad) [25]. Raw *C*_q_ values were calculated in Bio-Rad CFX manager software v.3.1 using automatic baseline and threshold settings. MiRNAs with coefficient of variation values > 4%, indicating high technical variability, were excluded from the analysis. Data were normalized using spike-in Cel-miR-39 and relative miRNA levels were expressed as log(2^−Δ*C*_q_^ * 10^6^).

### Statistical analysis

Statistical analysis was performed using SPSS 26.0. Normality of continuous variables was evaluated using Shapiro–Wilk test. Normally distributed data are expressed as mean ± standard deviation (SD), skewed variables as median (first to third quartile).

Unpaired data were compared using independent samples *t* test, Mann–Whitney U test or ANCOVA as appropriate. Pearson’s chi-squared test or Fisher’s exact test were used for comparison of categorical variables as appropriate.

Correlation among miRNAs, vascular measurements, OS and systemic inflammation was assessed using Pearson correlation analysis. To investigate the independent association between miRNAs and vascular function, multivariate linear regression analysis was performed, correcting for variables that are risk factors for PE (age and parity) or vascular function (age). A two-tailed *P*<0.05 was considered significant.

## Results

### Patient characteristics

Characteristics of HP and PE women are summarized in Table 1. According to the definition of PE, blood pressure was significantly higher in PE patients (*P*<0.05), which resulted in high percentages of antihypertensive treatment. The differences in birth weight were due to differences in gestational age at delivery (*P*<0.05). Platelet count was lower in PE compared with HP (*P*<0.05).

**Table 1:** Patient characteristics

	PE (*n*=24)	HP (*n*=30)	*P*-value
Age (years)	28.5 (26.7–30.9)	29.2 (27.4–32.5)	0.330
BMI at delivery (kg/m^2^)	29.1 ± 4.3	27.6 ± 4.1	0.241
SBP third trimester (mmHg)	152.6 ± 12.6	126.7 ± 12.3	<0.001
DBP third trimester (mmHg)	91.4 ± 7.6	74.3 ± 8.1	<0.001
MAP third trimester	111.8 ± 8.4	91.8 ± 8.9	<0.001
Nulliparous (*n*,%)	19 (79%)	19 (63%)	0.140
Gestation at delivery (weeks)	29.5 (28–31)	39.5 (39–40)	<0.001
Birth weight (g)	1244.2 ± 357.5	3513.8 ± 375.2	<0.001
Smoking (*n*, %)	3 (12.5%)	0 (0%)	0.082
Oral antihypertensive (*n*, %)	22 (92%)	0 (0%)	<0.001
Intravenous antihypertensive (*n*, %)	11 (46%)	0 (0%)	<0.001
Platelet count (× 10^9^/l)	169.4 ± 58.8	208.0 ± 58.2	0.024
Proteinuria (mg/24 h)	1985.0 (1061.0–3700.0)	NA	NA
Serum creatinine (mg/dl)	0.79 ± 0.1	NA	NA
AST (U/l)	43.5 (24.5–106.3)	NA	NA
ALT (U/l)	45.0 (18.0–139.8)	NA	NA

Data are expressed as mean ± SD, as median (first to third quartile) or as total number (*n*, %). Abbreviations: BMI, body mass index; DBP, diastolic blood pressure; MAP, mean arterial pressure; NA, not applicable; SBP, systolic blood pressure.

### Non-invasive measurement of peripheral vascular function

Table 2 shows that both macro- and microvascular endothelial function and arterial stiffness were impaired in PE compared with HP (all *P*<0.05). L-FMC and mFMD provide insight into the ‘resting’ endothelial capacity in contrast with the gold standard FMD, reflecting endothelial nitric oxide bioavailability [6].

**Table 2:** Vascular function measurements

		PE	HP	*P*-value
**Endothelial function**	FMD	5.82 ± 4.72	9.82 ± 4.76	0.008
	mFMD	6.37 ± 4.10	12.21 ± 5.55	0.001
	L-FMC	0.22 (−0.82 to 1.48)	−1.68 (−3.40 to −0.03)	0.016
	RHI	2.3 (2.03–2.4)	1.4 (1.3–1.65)	<0.001
**Arterial stiffness**	CF-PWV	7.58 (7.00–7.98)	6.11 (5.50–6.39)	<0.001
	AIx75	22.73 ± 7.62	2.79 ± 11.42	<0.001

Vascular function measurements in PE (diagnosis) vs HP (33–36 weeks (average: 34.7 weeks)). Data are expressed as mean ± SD or as median (first to third quartile).

### Plasma levels of VEGF-related miRNA, OS and systemic inflammation

In PE patients, levels of miR-16 were lower and miR-200c levels were higher compared with HP (*P*<0.05). Superoxide and MPV were higher in PE compared with HP (*P*<0.05), but no significant difference in NLR was observed (Table 3).

**Table 3:** Plasma miRNA expression (log(2^−Δ*C*_q_^ * 10^6^)), OS and systemic inflammation

	PE (*n*=24)	HP (*n*=30)	*P*-value
miR-16	5.53 ±0.36	5.84 ± 0.61	0.024
miR-29b	0.59 ± 0.47	0.87 ± 0.69	0.091
miR-126	3.59 ± 0.38	3.69 ± 0.56	0.465
miR-155	2.04 ± 0.46	2.24 ± 0.59	0.184
miR-200c	1.34 ± 0.57	0.97 ± 0.68	0.039
Superoxide (µM)	295.34 (211.87–383.30)	174.64 (129.70–237.56)	0.004
NLR	5.8 (3.53–8.05)	4.70 (3.55–5.23)	0.119
MPV (fl)	8.85 (8.50–10.25)	8.30 (7.70–9.05)	0.007

Blood sample was used to assess miRNA expression, OS and systemic inflammation in PE (diagnosis) vs HP (22–36 weeks (average: 25.8 weeks)). Data are expressed as mean ± SD or as median (first to third quartile).

### Associations between miRNAs and platelets (in HP and PE), and miRNAs and proteinuria, creatinine, AST and ALT (PE only)

No correlations among miRNAs, platelets, proteinuria, creatinine, AST and ALT were observed (all *P*>0.05, data not shown).

### Association between miRNAs and vascular function

**Figure 2:**
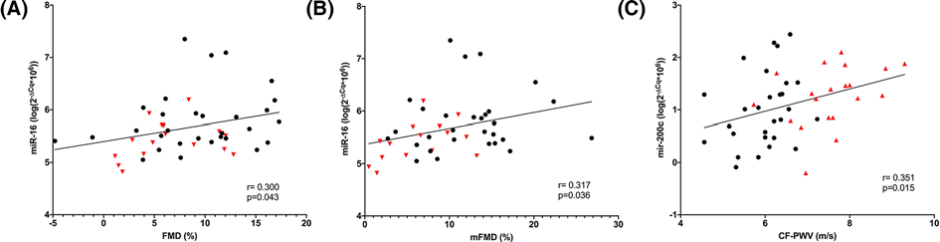
Pearson correlations between miRNAs and vascular function (**A**) miR-16 is significantly correlated with FMD (*P*=0.043). (**B**) miR-16 is significantly correlated with mFMD (*P*=0.036). (**C**) miR-200c is significantly correlated with CF-PWV (*P*=0.015). Black dots: healthy pregnant women. Red triangles: preeclamptic women.

In addition to this differential regulation in PE, a correlation of miR-16 and miR-200c with vascular function measurements was observed, as shown in Figure 2. Higher miR-16 levels were related to a better endothelial function (correlation with FMD r = 0.300, *P*=0.043 and mFMD r = 0.317, *P*=0.036). In contrast, higher miR-200c levels were related to increased arterial stiffness (correlation with CF-PWV r = 0.351, *P*=0.015). None of the miRNAs were correlated with OS or systemic inflammation (*P*>0.05, data not shown). To investigate whether miRNAs were independently associated with impaired vascular function, multivariate linear regression analysis, adjusting for age and parity, was performed. After adjustment, miR-16 remained independently associated with FMD (β 2.771, 95% C.I.: 0.023–5.519, *P*=0.048) and mFMD (β 3.401, 95% C.I.: 0.201–6.602, *P*=0.038). Likewise, miR-200c remained independently associated with CF-PWV (β 0.513, 95% C.I.: 0.034–0.992, *P*=0.036).

## Discussion

In this prospective case–control study, an innovative approach to the dismantling of the VEGF pathway in PE was applied by studying circulating levels of miRNAs that are known to influence VEGF expression in a post-transcriptional manner. We observed significantly lower circulating miR-16 levels and higher miR-200c levels in PE patients, correlating with impaired endothelial function and arterial stiffness, respectively.

Pregnancy is known to cause massive cardiovascular changes, starting as early as the first trimester. This hyperdynamic circulation, characterized by an increase in cardiac output, intravascular volume and systemic compliance, is necessary to meet the metabolic demands of pregnancy. In PE, this vascular adaption is impaired due to a pre-existing subclinical vascular dysfunction, together with a dysfunctional angiogenesis at the placental site. With VEGF as the central player, placental angiogenesis starts very early in pregnancy and optimal vascular resistance at the placental site is essential for HP. In the beginning of the normal placentation process, VEGF concentration is increased to avoid early trophoblast invasion in order to maintain an essential hypoxic state. After 8–10 weeks of gestation, VEGF concentration decreases resulting in trophoblast invasion of the spiral arteries mandatory for normal placentation. In pregnancies destined to develop PE, this early physiologic drop in VEGF concentration appears to be absent, causing the placenta to become ischemic [20]. This ischemic placenta secretes anti-angiogenic factors and OS in the circulation harming the vascular wall, resulting in impaired endothelial and arterial function as previously described [18].

Where decreased VEGF levels are repeatedly shown in term placental tissue after PE [7], data concerning circulating VEGF levels vary, with some studies showing an increase in circulating VEGF [14], while other report decreased or unchanged levels in PE [7]. Therefore, VEGF cannot be considered a reliable predictive marker for PE. Furthermore, placental miRNAs have been extensively studied. They are known to regulate placental development and abnormal placental miRNA expression has been associated with PE [4]. MiR-16, miR-29b, miR-126 and miR-200c, have all been shown to regulate the *placental* expression of VEGF.

In the present study, a panel of five miRNAs targeting the VEGF pathway, was selected. Whereas miR-16, miR-29b and miR-200c all directly target the 3′ UTR of VEGF mRNA, miR-126 and miR-155 indirectly influence VEGF expression. There are several lines of evidence that these five miRNAs indicate direct or indirect involvement in PE.

MiR-16 can significantly inhibit cell proliferation and invasion, promote cell apoptosis and suppress cell cycle progression. It also reduces inflammation, suppressing the secretion and mRNA expression of pro-inflammatory factors, such as interleukin (IL)-6 and tumor necrosis factor-α (TNF-α), and increased miR-16 enhances the secretion and mRNA expression of the anti-inflammatory factor IL-10. In literature, miR-16 is known to regulate placental angiogenesis by directly modulating the expression of VEGF and other angiogenic factors [9,21–23].

MiR-29b is involved in cell proliferation, differentiation and apoptosis. In pregnancy, miR-29b may play an essential role in trophoblast invasion through diminishing the activation of FAK phosphorylation. Dysregulated miR-29b disturbs proper trophoblast cell invasion, survival and angiogenesis, which may lead to the onset of PE [24].

MiR-126 influences the VEGF pathway through inhibition of Spred1 and lower levels of placental miR-126 in PE were associated with decreased VEGF placental expression [25].

MiR-155 down-regulates SOCS1, which leads to the activation of the JAK2/STAT3 signaling pathway and increases VEGF-A. MiR-155 also modulates endothelial nitric oxide synthase (eNOS) expression and inhibits Flt-1 expression [26].

MiR-200c plays a role in EC dysfunction and the formation of OS. Firstly, it inhibits ZEB1 protein, which induces apoptosis and senescence of ECs. Secondly, it disrupts the autoregulatory loop existing among Sirtuin1 (SIRT1), eNOS and FOXO1, causing NO decrease and OS increase [27]. MiR-200c not only regulates the expression of VEGF and its receptor Flt1 in endometrial cell lines, but also induces apoptosis of trophoblasts through inhibition of the Wnt/β-catenin pathway in a rat model of PE [27,28]. In the following paragraphs we will outline our findings in relation to the current evidence.

Here, we show a down-regulation of circulating miR-16 in early PE and reduced miR-16 levels were directly related to the impaired peripheral endothelial vasodilation as observed in PE. MiR-16 was previously observed to be overexpressed in placental tissues affected with severe PE where it inhibits VEGF expression, which results in inhibition of migration of ECs and trophoblasts, ultimately limiting angiogenesis in placenta [9,17]. A study by Wu et al. brought the evidence of circulating miR-16 down-regulation in PE using miRNA microarrays. However, these findings could not be confirmed using RT-qPCR in a small (*n*=19) group of late PE patients in that study [29]. The miR-16–VEGF interaction is auto-regulatory: miR-16 inhibits VEGF gene expression and [9,17] VEGF itself induces expression of miR-16 [21]. Furthermore, serum progesterone concentrations are decreased in PE and breast cancer research has shown that progestin down-regulates miR-16 expression [22]. This might be an additional mechanism contributing to the increased miR-16 levels observed in PE placenta compared with normal pregnancy.

In contrast with miR-16 levels, circulating miR-200c levels were higher in PE compared with HP and higher miR-200c was related to increased CF-PWV. Increased arterial stiffness has previously been observed in PE [6] and is related to higher cardiovascular risk later in life [30]. MiR-200c directly inhibits the expression of VEGF and Flt1, and it induces apoptosis of trophoblasts [28]. The biological significance of VEGF and its receptors (Flt1 and VEGFR1) in angiogenesis is well established. To the best of our knowledge, only two studies previously showed differential miR-200c expression in PE placenta. One study showed up-regulated miR-200c in both early and late onset PE placenta (*n*=19) compared with HP (*n*=23) [31].

The main strength of the present study is the focus on early PE, since discrimination between the different phenotypes of PE (early vs late) is important as they may involve different sets of miRNA expression. However, gestational age at sample collection was slightly, but significantly different between PE and HP, which could have influenced the miRNA levels as these also might change with advancing HP [3]. Despite this difference in gestational age, the relationship between early PE and two important miRNAs of the VEGF pathway suggest critically functioning roles of these miRNAs in the pathophysiology of this pregnancy-related disease. Second, we quantified circulating miRNAs instead of placenta derived miRNAs as we believe circulating miRNAs are more valuable in the prediction of PE or vascular dysfunction. Due to their stability in plasma, the use of miRNAs in the prediction, diagnosis and/or monitoring of PE could have a great potential.

## Conclusions

The VEGF pathway is essential in the placentation process and dysregulation of it contributes to the development of PE. In this case–control study, we investigated whether five circulating miRNAs, known to target the VEGF pathway, are related to vascular function, OS and systemic inflammation in PE vs HP. We observed down-regulated plasma levels of miR-16, associated with impaired endothelial function, and up-regulated levels of miR-200c, correlated with increased arterial stiffness in early PE. Hence, these findings suggest a post-transcriptional dysregulation of the VEGF pathway in PE and identify miR-16 and miR-200c as possible diagnostic biomarkers for PE. Whereas plasma levels of miR-200c are in-line with evidence on placental samples, the findings on circulating miR-16 are in contrast with evidence in placental samples. Therefore, our findings are hypothesis-generating and need confirmation in further experiments.

## Perspectives

- PE is a severe pregnancy complication with a crucial role of pre-existent maternal endothelial dysfunction leading to impaired placentation. The VEGF pathway and its post-transcriptional modification by miRNAs seems essential in the placentation. However, circulating VEGF levels are not consistently associated with PE, hence our approach to study circulating miRNAs known to regulate VEGF expression in relation to PE.
- In the present study, we demonstrate that circulating miR-16 levels are decreased in preeclamptic patients, and lower levels were associated with an impaired endothelial-dependent vasodilatation. Circulating miR-200c levels are increased in PE and are related to arterial stiffness.
- Several studies show differential miRNA expression in *placental* tissue of preeclamptic patients. With regard to prediction, diagnosis and monitoring of PE, *circulating* miRNA emerge as promising biomarkers.

## Data Availability

We declare that the materials described in the manuscript are available from the corresponding author upon request. All relevant raw and edited data will be freely accessible to any scientist for non-commercial purposes, without breaching participant confidentiality.

## Abbreviations

AIx75: heart rate corrected augmentation index
ALT: alanine transaminase
ANCOVA: analysis of covariance
AST: aspartate aminotransferase
CF-PWV: carotid-femoral pulse wave velocity
C.I.: confidence interval
EC: endothelial cell
EDTA: ethylenediaminetetraacetic acid
eNOS: endothelial nitric oxide synthase
FMD: flow-mediated dilatation
FOXO1: forkhead box protein O1
HP: healthy pregnancy
IL: interleukin
L-FMC: low-flow mediated constriction
mFMD: modified FMD
miRNA: microRNA
MPV: mean platelet volume
NLR: neutrophil to lymphocyte ratio
OS: oxidative stres
PE: preeclampsia
PlGF: placental growth factor
RT-qPCR: reverse transcription quantitative polymerase chain reaction
sFlt-1: soluble fms-like tyrosine kinase-1
SOCS1: suppressor of cytokine signaling 1
VEGF: vascular endothelial growth factor
